# The Increase in the Peroxidase Activity of the Cytochrome *C* with Substitutions in the Universal Binding Site Is Associated with Changes in the Ability to Interact with External Ligands

**DOI:** 10.3390/ijms25158237

**Published:** 2024-07-28

**Authors:** Rita V. Chertkova, Ilya P. Oleynikov, Alexey A. Pakhomov, Roman V. Sudakov, Marina A. Semenova, Alexander M. Arutyunyan, Vasily V. Ptushenko, Mikhail P. Kirpichnikov, Dmitry A. Dolgikh, Tatiana V. Vygodina

**Affiliations:** 1 Shemyakin-Ovchinnikov Institute of Bioorganic Chemistry, Russian Academy of Sciences, 117997 Moscow, Russia; alpah@mail.ru (A.A.P.); marinaapbch@mail.ru (M.A.S.); kirpichnikov@inbox.ru (M.P.K.); dolgikh@nmr.ru (D.A.D.); 2 A.N. Belozersky Institute of Physico-Chemical Biology, M.V. Lomonosov Moscow State University, Leninskie Gory 1, Bld. 40, 119992 Moscow, Russiasudakovromvlad@gmail.com (R.V.S.); alarut@belozersky.msu.ru (A.M.A.); ptush@belozersky.msu.ru (V.V.P.); 3 N.M. Emanuel Institute of Biochemical Physics, Russian Academy of Sciences, 119334 Moscow, Russia; 4 Biology Department, M.V. Lomonosov Moscow State University, 119899 Moscow, Russia

**Keywords:** cytochrome *c*, heme, universal binding site, electron transport chain, methionine 80, ligand binding, peroxidase activity, molecular dynamic simulations, magnetic circular dichroism

## Abstract

Cytochrome *c* (CytC), a one-electron carrier, transfers electrons from complex *bc*_1_ to cytochrome *c* oxidase (CcO) in the electron-transport chain. Electrostatic interaction with the partners, complex *bc*_1_ and CcO, is ensured by a lysine cluster near the heme forming the Universal Binding Site (UBS). We constructed three mutant variants of mitochondrial CytC with one (2Mut), four (5Mut), and five (8Mut) Lys->Glu substitutions in the UBS and some compensating Glu->Lys substitutions at the periphery of the UBS for charge compensation. All mutants showed a 4–6 times increased peroxidase activity and accelerated binding of cyanide to the ferric heme of CytC. In contrast, decomposition of the cyanide complex with ferrous CytC, as monitored by magnetic circular dichroism spectroscopy, was slower in mutants compared to WT. Molecular dynamic simulations revealed the increase in the fluctuations of C_α_ atoms of individual residues of mutant CytC compared to WT, especially in the Ω-loop (70–85), which can cause destabilization of the Fe…S(Met80) coordination link, facilitation of the binding of exogenous ligands cyanide and peroxide, and an increase in peroxidase activity. It was found that only one substitution K72E is enough to induce all these changes, indicating the significance of K72 and the Ω-loop (70–85) for the structure and physiology of mitochondrial CytC. In this work, we also propose using a ferro-ferricyanide buffer as a substrate to monitor the peroxidase activity of CytC. This new approach allows us to determine the rate of peroxidase activity at moderate (200 µM) concentrations of H_2_O_2_ and avoid complications of radical formation during the reaction.

## 1. Introduction

Cytochrome *c* (CytC), a small (only 104 amino acid residues) alkaline protein, located in the intermembrane space of mitochondria (IMS), transfers electrons from complex *bc*_1_ to cytochrome oxidase, and it is the only water-soluble mobile element among membrane-bound enzyme complexes of the electron transport chain (ETC). In addition to its main function as an electron carrier, cytochrome *c* has a number of alternative functions in the cell, the most important of which is a proapoptotic one: participation in apoptosis signaling pathways including peroxidation of cardiolipin, permeabilization of mitochondrial membrane, and apoptosome formation, see reviews [1,2]. Peroxidation of cardiolipin by CytC was discovered by Kagan’s group in the early nineties, and the key role of this event in the induction of apoptosis has been elucidated in many reviews [1,2,3,4,5].

Peroxidase reaction catalyzed by heme peroxidases consists of a two-stepwise single electron transfer to the heme-bound peroxide, which is finally reduced to water. Each step is associated with the formation of the appropriate intermediate, called Compound I and Compound II, correspondingly with a very high redox potential (ca +1 V), which allows them to oxidize high-potential substrates like phenols, phospholipids, etc. (for details of mechanism see [6,7,8]). This inherent feature of peroxidase reaction actually determines the main signs of apoptosis development: peroxidation of cardiolipin and permeabilization of mitochondrial membrane, leading to the release of CytC into the cytosol, initiating apoptosome formation [2,3,9].

**Figure A1:**
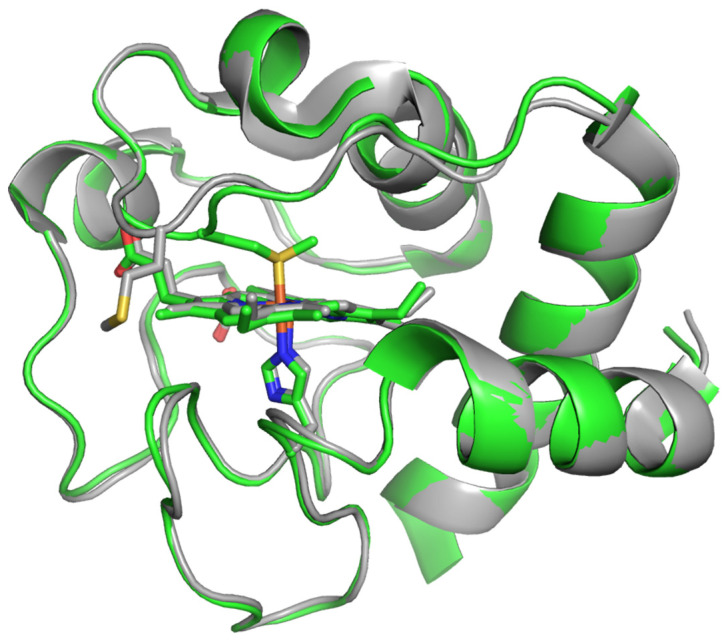
The crystal structures of 6-coordinated CytC WT from horse heart (PDB ID:1HRC, green) and 5-coordinated yeast CytC with K72A mutation (PDB ID: 4MU8, gray, see [20]) are aligned along α-helices and superimposed. The region around heme iron is shown. The heme, His18, and Met80 are presented as stick models (porphyrin rings lying perpendicular to the plane of the figure in the middle). Axial ligands are marked by yellow (sulfur of M80) and blue (nitrogen of H18) and corresponding Fe…S(M80) and Fe…N(H18) coordination bonds are indicated by orange sticks.

The active center of CytC is formed by hemoporphyrin type *c* containing a central Fe atom tightly ligated by nitrogen of His18 and weakly by sulfur of Met80 [10] (see also Figure A1 in Appendix A). There is not enough space for peroxide in the heme pocket, but it is formed upon the development of apoptosis. Under the influence of various apoptosis-inducing factors, the CytC function switches from an electron transport protein in the ETC to the function of a proapoptotic factor, which starts by enhancing the CytC–cardiolipin interaction. According to a widespread hypothesis, the formation of the CytC–cardiolipin complex results in the cleavage of the hydrogen bond between the residues His26 and Pro44 (site C) [11,12], and the coordination bond heme Fe…S(M80), which changes the conformational structure of CytC and, by up to ten times, increases its peroxidase activity with the simultaneous loss of the ability to transfer electrons [13]. All these events precede the initiation of a cascade of reactions leading to cell apoptosis [3,4] and make heme available for interaction with hydrogen peroxide and other ROS.

In addition to site C, presumed to be involved in the interaction with cardiolipin, CytC has two more binding sites, A and L, consisting mainly of positively charged lysine residues from the universal binding site (UBS), which provide the correct spatial orientation of CytC and electrostatic interaction with ETC partners *bc*_1_ and CcO: site A—residues Lys72, Lys73, Lys86, Lys87, and Arg91 and site L—residues Lys22, Lys25, Lys27, and His33 (the latter also acquires a positive charge in an acidic environment) [14]. It is also not excluded that these sites may be responsible for electrostatic interactions within the CytC/cardiolipin complex, which do not cause critical changes in the tertiary structure of the protein [15].

Previously, we constructed and studied mutant variants of CytC with substitutions of Lys residues from UBS by Glu. All these mutant CytCs lack electron transport activity [16]. In the previous article [17], the aim was to select the effective sensor for superoxide among CytC mutants, rendering it inactive in ETC; three variants with K8E/K27E/E62K/E69K/K72E/K86E/K87E/E90K (8Mut), K8E/E69K/K72E/K86E/K87E (5 Mut), and E69K/K72E (2Mut) were made. It was found that substitutions of one (2Mut), four (5Mut), and five (8Mut) Lys->Glu in the UBS, and Glu->Lys substitutions at the periphery of the UBS for charge compensation one (2Mut), one (5Mut), and three (8Mut), converted CytC to a completely (5Mut, 8Mut) or partially (2Mut) inactive electron transporter in ETC but retained its antioxidant properties as radical scavenger. The study of physico-chemical characteristics of mutant CytC has revealed that 5Mut and 8Mut with a similar number (five in 8Mut and four in 5Mut) of K/E replacements in the UBS but a different amount of E/K replacements (three in 8Mut and only one in 5Mut) compensating the surface charge demonstrated a quite different effect of mutations on the protein structure. A significant increase in the amplitude of the CD spectrum of 5Mut in comparison with the CD spectra of CytC 2Mut, 8Mut, and CytC WT, which sufficiently coincide (Figure 7 in [17]), as well as the aggregation tendency of CytC 5Mut, revealed by the dynamic light scattering (Figure 8 in [17]), indicate a significant effect of charge imbalance on the overall structure of the protein globule. As a result, 8Mut with no activity in ETC, but retaining most of its physico-chemical properties and antioxidant activity as a radical scavenger, was proposed to be a good candidate to measure superoxide generation [17].

Our current study showed that the same three mutants have a 4- to 6-fold enhanced peroxidase activity compared to WT CytC in solution in the absence of cardiolipin or membranes and the increased ability to interact with exogenous ligands, which we associated with the increased distance between heme iron and S(Met80), resulting in the appearance of free space in the heme pocket for peroxide and other exogenous ligands.

## 2. Results

### 2.1. Peroxidase Activity of Mutant CytC Forms

As mentioned in the Introduction, in the normal state, CytC virtually does not catalyze the peroxidase reaction, so its registration is a separate task. There are several known methods for determining the peroxidase activity of CytC, but they all have certain significant drawbacks, which include the fact that the substrate used forms a radical during the reaction [18], or the addition of too high concentrations of peroxide [19,20,21], or the impossibility of quantitative assessing the rate of peroxidase reaction [3,22]. The available methods will be discussed in more detail in Section 3.

In our work, it was proposed to use a fundamentally new substrate to determine peroxidase activity, which is a mixture of ferro- and ferricyanide, the ratio of the components of which allows us to flexibly change its redox potential. In the case of CytC, the optimal ratio was ferro-/ferri- 6:1 (Em 370 mV), at which the rate of the peroxidase reaction involving CytC was significantly higher than the rate of ferrocyanide oxidation with peroxide (control). The remaining reaction registration parameters (pH 7.1, CytC concentration (3 µM) within the range of values at which the rate linearly depends on the CytC concentration) were taken from [18], which is closest in registration conditions to our study, but uses ABTS as a substrate, which forms a radical during oxidation. The oxidation of ferrocyanide to ferricyanide is a one-electron reaction not associated with the formation of radicals, the rate of which is estimated spectrophotometrically using ∆ε_420–500_ 1040 M^−1^ cm^−1^ for ferricyanide. The iron chelator 0.2 mM EDTA was added to the buffer to eliminate the influence of impurity iron.

**Figure 1:**
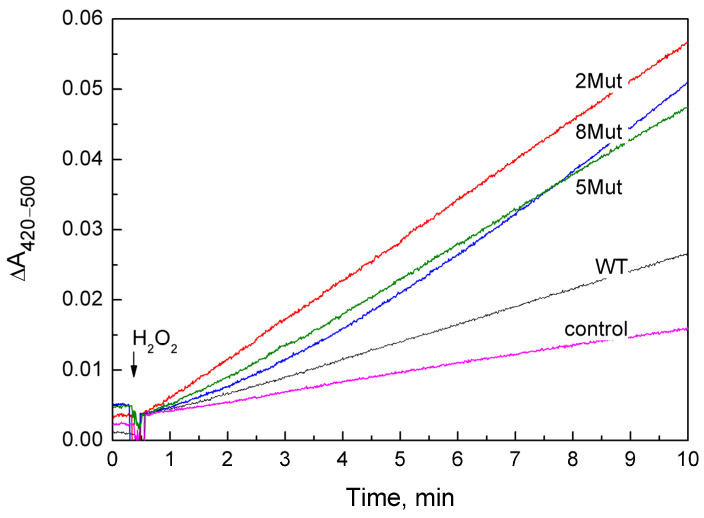
Peroxidase activity of CytC WT (black curve) and mutant CytC: 2Mut (red curve), 5Mut (green curve), and 8Mut (blue curve), recorded by the difference in absorption at 420–500 nm, corresponding to the peroxidation of ferrocyanide to ferricyanide. Control (magenta)—oxidation of ferrocyanide in the absence of CytC. CytC (3 µM), ferrocyanide 1.2 mM, ferricyanide 0.2 mM were added to the measurement medium (buffer 20 mM MOPS pH 7.1, 50 mM KCl, 0.2 mM EDTA). The reaction was initiated by adding 200 µM H_2_O_2_.

A set of kinetic curves used to determine the peroxidase activity of CytC WT and its mutant forms is presented in Figure 1. The curves with the reaction rate closest to the average were selected. In most cases for CytC variants, it was convenient to determine the rate, since the curves are almost linear, with the exception of CytC 8 Mut, in which case the rate was determined from the final linear section. The obtained speed values are given in Table 1 both as the rate of ferricyanide formation (µM/min) (the average value obtained in 3–4 independent experiments is given, indicating the deviation), and in the turns of the enzyme per minute, when the rate of the reaction minus the control was divided by the concentration of CytC. Notably, the rate of the peroxidase reaction catalyzed by mutant cytochromes is 4–6 times higher than that of CytC WT, and only one substitution (K72E) in CytC 2Mut is sufficient for activation, since the E69K substitution simply compensates for changes.

The rates of ferrocyanide peroxidation by recombinant CytC WT (0.31 min^−1^) and the commercial CytC preparation isolated from the horse heart (0.36 ± 0.2 min^−1^) were comparable, but turned out to be almost four times higher than the rate (0.08 min^−1^) recalculated from data from [18]. This may be due to the greater efficiency of the negatively charged ferrocyanide as a substrate compared to the uncharged ABTS used in [18].

### 2.2. Effect of Mutations in UBS on Cyanide Binding to CytC

The catalytic cycle of heme peroxidases begins with the coordination of peroxide to the ferric heme. To determine the reason for the acceleration of peroxidase activity of mutants, it was necessary to find out how mutations in UBS affect the binding of exogenous ligands to heme iron. The binding of exogenous ligands is an important property of hemoproteins. This mainly concerns enzymes that interact with oxygen: globins (myoglobin, hemoglobin) and CcO. One of the axial heme iron ligands of these proteins is weak and can be replaced by oxygen, see review [23]. Ligand binding studies provide important information about the structure of the heme center. For CytC, interaction with ligands is less common, but possible. As mentioned earlier, the axial ligands of heme iron in CytC are N…His18 and S…Met80. The bond with sulfur is weaker and under some conditions (such as alkalization), the length of the Fe…S bond can increase, allowing exogenous ligands to bind to the heme. In this work, we investigated the effect of mutations in UBS on the ability of CytC to bind cyanide, a universal ligand of heme iron, which binds to CytC in both the oxidized and reduced states.

#### 2.2.1. Acceleration of Cyanide Binding to Oxidized Forms of Mutant CytC as an Indicator of Changes in the Strength of Interaction between Heme Iron and S(Met80)

**Figure 2:**
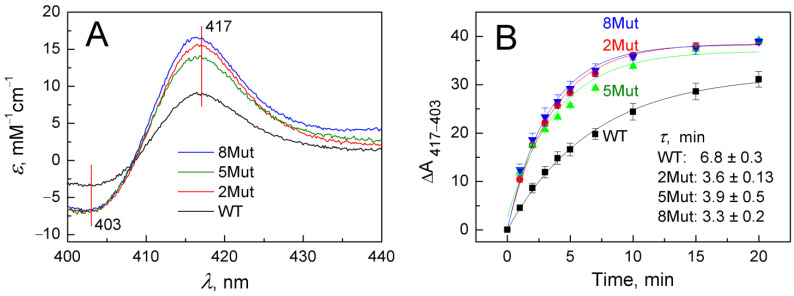
Long-wavelength spectral shift in the Soret absorption band caused by cyanide binding to oxidized CytC WT and its mutant variants: (**A**)—Difference spectra of CytC (vs. oxidized form) recorded in the base buffer in 3 min after adding cyanide (5 mM) and normalized to protein concentration in the sample: CytC WT (black spectrum), CytC 2Mut (red spectrum), CytC 5Mut (green spectrum), and CytC 8Mut (blue spectrum). (**B**)—Time dependence of spectral changes in CytC upon binding to cyanide, the color of the curves corresponds to the color of the spectra in panel A.

As in the case of other hemoproteins, for example, CcO, cyanide binding to oxidized heme iron of CytC induces a long-wavelength shift in the absorption spectrum of the enzyme, which is more clearly visible in the corresponding difference spectra recorded versus the original oxidized form of the protein. Representative difference spectra indicating cyanide (5 mM) binding to heme iron at the beginning of the reaction (3 min after cyanide addition) are shown in Figure 2A. In the Soret band, they have a typical symmetrical butterfly shape with a minimum of 403 nm and a maximum of 417 nm. To study cyanide binding in detail was not the purpose of this study. We aimed only to demonstrate the differences in the ability of WT and its mutant forms to bind cyanide, which was clearly visible already at the initial stage. It can be seen (Figure 2A) that spectral changes induced in mutants (color spectra) develop much faster than in WT (black spectrum). The differences in the rate of cyanide binding are clearly observed in the kinetic traces presented in Figure 2B. Data points on the curves correspond to changes in the amplitudes of the spectra recorded in the Soret band over a time period elapsed after ligand addition and reflect the absorbance difference at 417 nm versus 403 nm normalized to the concentration of CytC. It can be seen that τ values for the formation of the cyanide complex in WT are approximately twice as high compared to 2Mut and 8Mut, which actually means that the rate is half less than in mutants. The τ value for 5Mut differs slightly less (1.7 times) from WT, and the data are less well approximated, which may be explained by the previously observed differences in the physico-chemical properties of this mutant due to the predominance of its negative charge due to unbalanced substitutions (five substitutions K/E and only one—E/K). Over time, the difference in amplitudes is gradually leveled out; however, in mutant forms (colored curves), the formation of the cyanide complex is virtually completed in 20 min (Figure 2B), while only 75–80% of the cyanide complex with CytC WT (black curve) is formed by this time.

#### 2.2.2. Deceleration of the Decay of the Cyanide Complex with the Reduced form of CytC, Caused by Mutations in UBS, Recorded by the MCD Method

**Figure 3:**
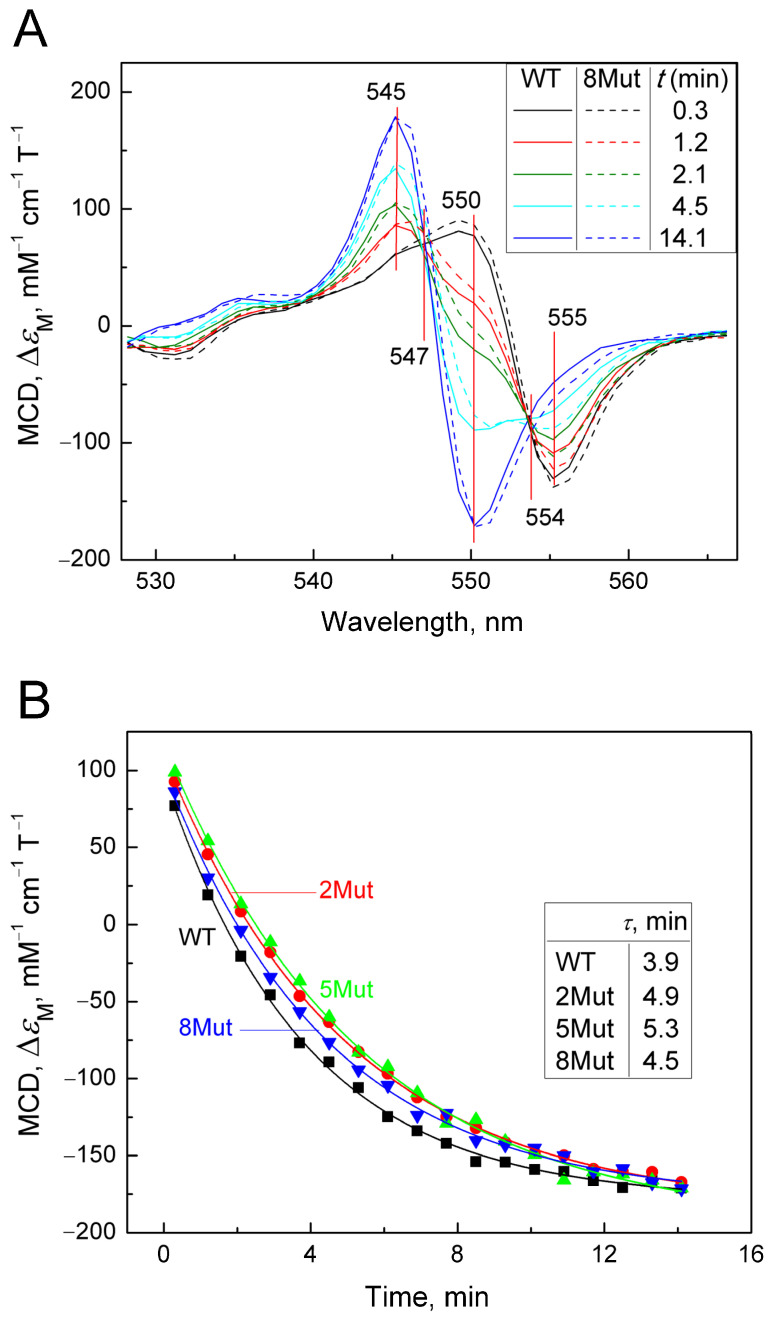

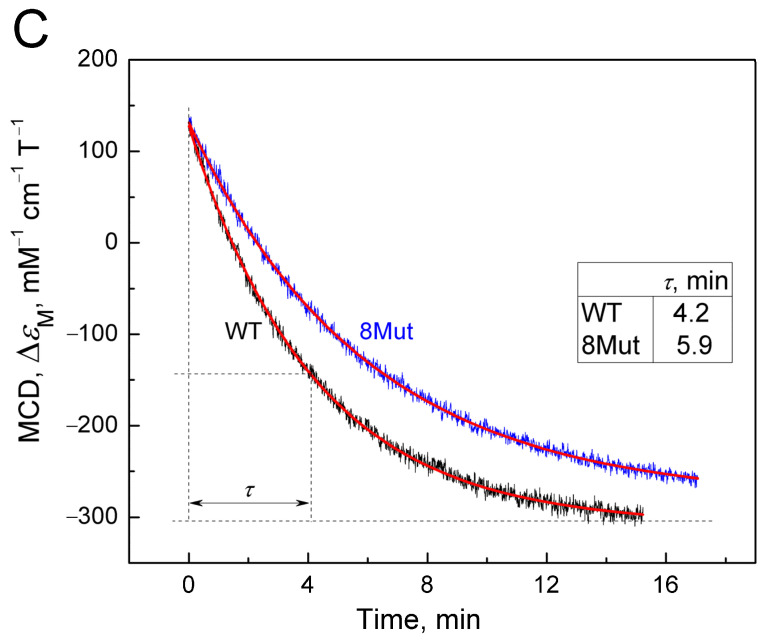
Degradation of the CytC^2+^-CN complex registered by MCD. MCD spectra were recorded in a 50 mM K-Pi/tris buffer (pH 8.7). Concentration of CytC (of WT and mutant forms) in the sample was around 25 µM. Additions: KCN 100 mM, dithionite as a powder: (**A**)—a set of absolute spectra (colored), reflecting the decomposition of the cyanide complex of the reduced CytC of WT (solid lines) and 8Mut (dotted lines) in time after the reduction of heme Fe with dithionite. Spectra were recorded at the following time intervals in minutes: 0.3 (black), 1.2 (red), 2.1 (blue), 4.5 (magenta), and 14.1 (green); (**B**)—kinetics of changes in the MCD signal at 550 nm, reconstructed from the spectra from panel A for CytC WT (black curve), 2Mut (red curve), 5Mut (green curve), and 8Mut (blue curve). The curves are theoretical curves drawn through experimental points; (**C**)—change in the MCD signal at 550 nm, recorded for CytC of WT (black curve) and 8Mut (blue curve) in the kinetic mode. The theoretical curves reflecting the exponential decay of the signal are shown in red.

**Figure A2:**
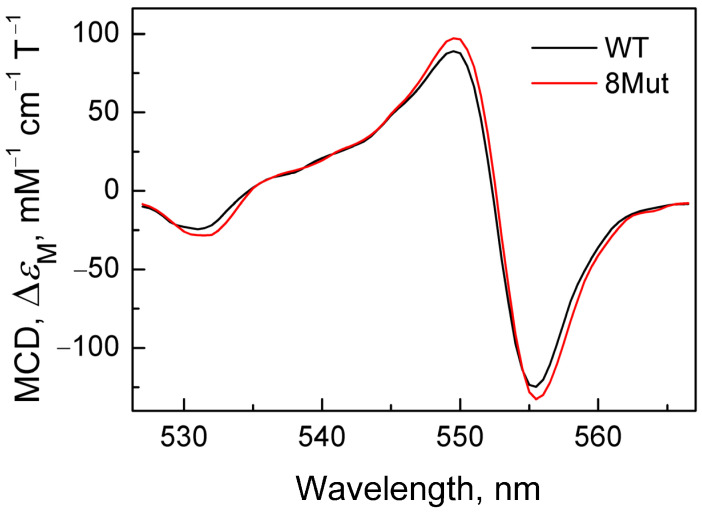
MCD spectra of cyanide complex with CytC^2+^ of WT (black) and 8Mut (red) reconstructed with Graph Work program based on the spectrum recorded within 20 s after dithionite addition (first recorded spectrum).

**Figure A3:**
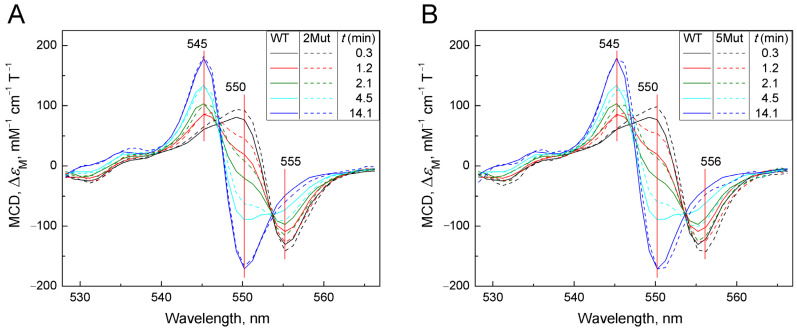
A set of absolute spectra (colored) reflecting the decay of the complex CytC^2+^-CN: (**A**)—CytC of WT (solid spectra) is compared with 2Mut (dotted spectra); (**B**)—CytC of WT (solid spectra) is compared with 5Mut (dotted spectra). MCD spectra were recorded in a 50 mM K-Pi/tris buffer (pH 8.7). Concentration of CytC in the sample was around 25 µM. Additions: KCN 100 mM, dithionite as a powder. Spectra were recorded at the following time intervals in minutes after addition of dithionite: 0.3 (black), 1.2 (red), 2.1 (blue), 4.5 (magenta), and 14.1 (green).

The method of magnetic circular dichroism (MCD), that is, dichroism in a forward and reverse magnetic field, is a powerful method for studying hemoproteins and CytC, in particular, as described in [24,25]. It is more sensitive to the state of heme iron than CD, which is commonly used to monitor the structure of the protein part (amounts of α-helices, β-sheets, and unstructured regions). The limitation of the method is that only the reduced form of the protein has a good MCD signal. Cyanide as a ligand of hemoproteins, in particular, CcO, is distinguished by the fact that it interacts with both the oxidized and reduced forms of heme iron, although with different affinities. In the case of CytC, however, the reduced form has a very rigid structure that does not allow the ligand to be located in the heme pocket. It is possible to obtain a complex of the reduced form of CytC with cyanide only using special techniques (low temperature, alkaline pH), and the complex is extremely unstable and disintegrates within a few minutes [26]. However, using various spectrophotometric methods, including methods for recording fast kinetics, it is possible to monitor the degradation process of the CytC^2+^-CN complex. Considering the large signal magnitude and good resolution of MCD, we decided to use this method to record the decay of the cyanide complex with the reduced form of CytC in order to assess the effect of mutations. To date, the MCD method has not been used for this, so the results obtained have a value. The complex was prepared as described in the Materials and Methods Section; that is, first a complex of oxidized CytC with cyanide was obtained, after which the heme was reduced with dithionite (for standardization of measurements, and the samples were pre-oxidized with substoichiometric concentrations of ferricyanide). The countdown of the complex decomposition time began after the addition of dithionite. Due to the rapidity of complex degradation, the MCD signal was recorded only in the direct field, as CD contribution in this case was minimal and could be neglected. Recording times were standardized and monitored using a stopwatch. Spectra were recorded approximately every minute. The MCD spectra of CytC WT and CytC 8Mut recorded in this way are shown in Figure 3A. The Graph Work program created in the laboratory to control the instrument made it possible to calculate the content of the cyanide complex in the measured sample at each time point based on the spectrum of free CytC^2+^ obtained at the end of the experiment and the spectrum of the CytC^2+^-CN complex, reconstructed on the basis of the first spectrum recorded 20 s after the addition of dithionite. The data obtained are presented in Table 2. The reconstructed spectrum of the CytC^2+^-CN complex (see Figure A2 in Appendix A) has a characteristic symmetrical shape with a maximum at 550 nm and a minimum at 555 nm. The reconstruction was carried out on the basis of restoring the complete symmetry of the spectrum recorded first in time (Figure 3A, black spectrum) and was close to it in shape. The series of spectra in Figure 3A shows the decay over time of the cyanide complex with CytC^2+^ of WT (solid lines) and of 8Mut form (dotted lines), which is characterized by the rapid disappearance of the minimum at 555 nm with the simultaneous transformation of the peak at 550 nm into the minimum of the final spectrum and the appearance of a new maximum at 545 nm. In both mutants and CytC WT, the process is completed in approximately 14 min with the formation of a symmetrical MCD spectrum of the free form of CytC^2+^ with a maximum at 545 nm and a minimum at 550 nm, which makes it possible to normalize the recorded spectra to the signal of free reduced CytC. In Figure 3A, one should pay attention to the fact that all spectra intersect at two isosbestic points at 547 nm and 554 nm (this is especially clearly visible for the point 547 nm). The presence of isosbestic points is usually interpreted as confirmation of the adequacy of the recorded process. It is clearly visible that the development of spectral changes characterizing the degradation of the cyanide complex with CytC 8Mut (dashed line) lags in time relative to CytC WT (solid line). A similar series of spectra characterizing the decomposition of cyanide complexes 2Mut and 5Mut in comparison with WT is shown in Figure A3A,B (in Appendix A). Figure 3A shows that maximal changes in the MCD spectra associated with the decomposition of cyanide complexes occur at 550 nm; therefore, we reconstructed the kinetic curves following the changes in the MCD signal at 550 nm for WT and mutants (2Mut, 5Mut, and 8Mut) based on the obtained series of spectra over time (Figure 3B). It can be noted that changes in the MCD signal at 550 nm associated with the breakdown of the CytC^2+^-CN complex occur more slowly in all mutants than in WT. Experimental points are well approximated by theoretical curves of exponential decay, which allow τ values corresponding to a half-way reaction progressing to be calculated. It can be seen that τ values are higher for all mutants compared to WT.

To confirm the differences in the rate of the CytC^2+^-CN complex decay in the wild type and mutants, the decay of the cyanide complex with 8Mut and WT CytC^2+^ was separately recorded in a kinetic mode as a change in the MCD signal at 550 nm over time (Figure 3C). It can be seen that the decomposition of the cyanide complex with reduced CytC 8Mut noticeably lags behind WT in time, and for these curves, the effect is even more obvious: τ value for 8Mut (5.9 min) is 1.4 times higher compared to WT (4.2 min). Unfortunately, it was not possible to obtain such data for other mutants due to the large consumption of material in the experiments.

The Graph Work program, which controls the dichrograph for recording MCD spectra, allows us to calculate the content of individual components in it. Using this program, the content (in %) of the cyanide complex 8Mut and WT was calculated at each moment of recording the spectra, presented in Table 2. The data presented in Table 2 are consistent with the data in Figure 3 and reflect the same trend of slowing down the decay of the CytC^2+^-CN complex caused by mutations.

### 2.3. Molecular Modeling of the Structure of CytC WT and Its Mutant Variants 2Mut, 5Mut, and 8Mut

As mentioned above, the possibility of placing a ligand in the CytC heme pocket depends on the strength of interaction between heme iron and Met80 sulfur [23]. The spectral indicator of the state of the Fe…S bond in oxidized CytC is the so-called charge transfer (CT) band at 695–698 nm, which, in all CytC mutants, as shown in the previous article [17], did not differ much from WT, although in recombinant cytochromes, it was slightly smaller (approximately 0.13 mM^−1^ cm^−1^) than that of a commercial preparation of CytC from horse heart (0.2 mM^−1^ cm^−1^). In general, the absorption spectra of the mutants were almost no different from the wild type in either the position or magnitude of the bands. To correctly assess the influence of the structural changes induced by mutations on the peroxidase activity and binding of exogenous ligands with the heme center, molecular dynamic methods were applied.

**Figure 4:**
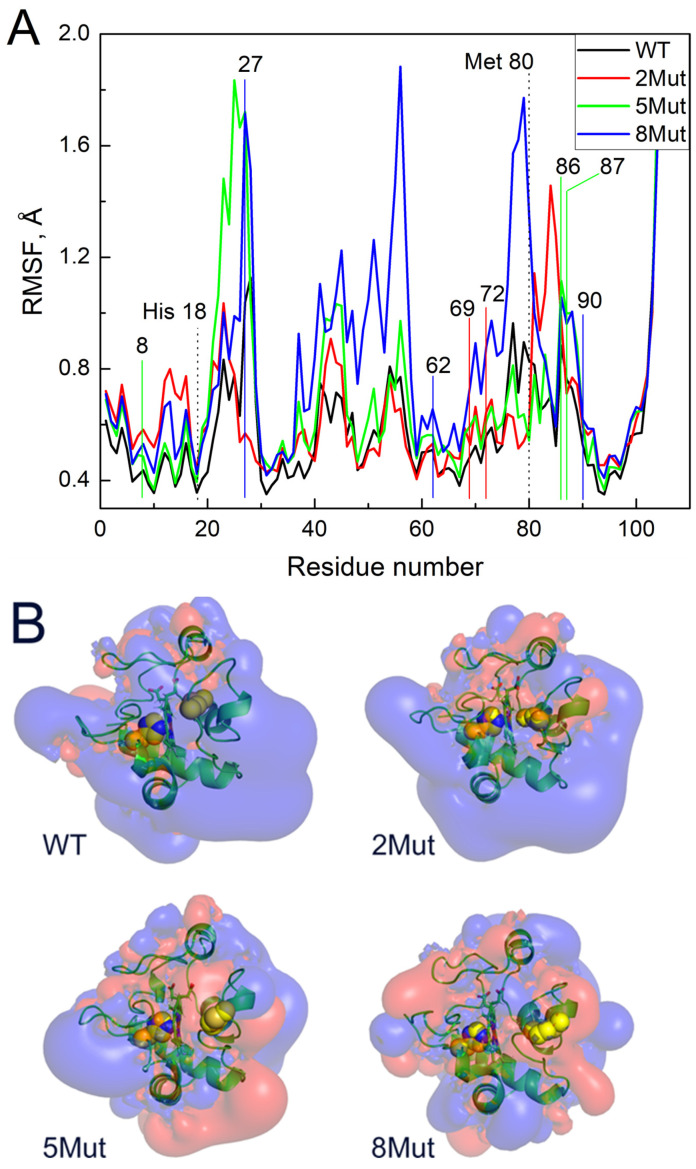
Molecular modeling of CytC WT and mutants calculated using the Gromacs software package (2023.5) [27]: (**A**)—root mean square fluctuations (RMSF) of C_α_ atoms of individual residues in CytC WT and mutants. The numbers on X-axis indicate the number of the residue and Y-axis reflects RMSF. The residues replaced in mutant forms are indicated by the numbers above the curves and for convenient visualization the replacements are marked by the colored lines corresponding to the color of the curve: 2 in 2Mut are marked by red, 3 additional in 5Mut—by green, 3 additional in 8Mut—by blue, the axial ligands of heme iron His 18 and Met80 are marked by dotted lines. (**B**)—isoelectric surfaces of horse CytC^3+^ (WT and mutant forms) shown from the side of the heme cavity located in the middle. Cavity in the center is divided by a heme plane that is perpendicular to the surface. The right part with Met80 as a weak axial heme ligand represents the place where exogenous ligands can bind while tightly bound left ligand His18 leaves no free space for ligands (Met80 and His18 side chains are shown as yellow spheres). Blue color reflects the isoelectric surface (+0.01 mV) while red areas correspond to the surface (−0.01 mV). The structures are taken from the 90 ns of MD trajectories.

Figure 4A shows the average fluctuations of individual residues (C_α_ atoms) in the CytC WT and mutant forms based on 350 ns MD simulations. The numbers of the replaced residues are indicated above the curves as well as the axial ligands of the heme iron His 18 and Met80. It is evident that 8Mut has the maximum number of fluctuations among all other residues studied (0–100), which correlates with its maximal peroxidase activity, and one of the most flexible is observed precisely in the region of residues 79K and 80M, the residues that affect the interaction of heme with ligands including peroxide. The maximum fluctuations of residues in 2Mut, which ranks second in activity in the peroxidase reaction, are shifted toward residues 84–86 at the end of the Ω-loop. At the same time, the flexibility of the residues in 5Mut in the region of the Ω-loop is minimal, like in WT in good correlation with its minimal peroxidase activity despite the general imbalanced structure identified previously [17]. It is noteworthy to mention that this part of the CytC molecule (residues 70–85 of the Ω-loop) is one of the most flexible. The second area of the increased mobility that is worth paying attention to is located near residues 42–56. And, again, the residues of 8Mut show maximum mobility in this region but fluctuations of the residues observed in CytC 5Mut are only a bit less, while in CytC 2Mut and especially in WT, they are much lower (Figure 4A). The last region of the increased flexibility of the residues in mutant CytC compared to WT is located in the region of residues 20–28 where the mobility of residues 27–28 in 8Mut and 5Mut > than in 2Mut > WT. It is also noteworthy that the most stable region of the structure with the minimum fluctuation is located in the H18 region of the axial heme iron ligand.

Figure 4B shows the results of modeling the equipotential electrostatic surfaces of the globule of oxidized CytC WT and mutants. One can see that the equipotential surface of WT is quite uniform and mostly positively charged (Figure 4B). Mutations make the electrostatics of the globule more diverse with large areas where a negative charge predominates (colored red), which leads to an increase in the mobility of amino acid residues, in particular the loop with Met80. Apparently, this facilitates the penetration of the external ligand into a hydrophobic pocket for heme binding. Particular attention should be paid to the differences in the hydrophobic pockets of the mutants. All mutants have more negatively charged areas in the region of the heme cavity than WT, but the cavity itself is less closed by side loops, especially in 5Mut and 8Mut, which may play a decisive role in facilitating ligand binding. This observation is consistent with the fact that 8Mut binds cyanide faster than WT and even other mutants and has the highest peroxidase activity. 5Mut has the largest negatively charged surface area in the heme cavity. The cyanide and deprotonated peroxide bind as an anion, so the negatively charged surface of the pocket will interfere with ligand binding. Indeed, of all the mutants, 5Mut binds cyanide the worst and has the lowest peroxidase activity.

Classic peroxidases have a five-coordinate heme, where the sixth (distal) site is vacant or occupied by loosely bound water and can easily bind external peroxide [6,21]. Introduction of peroxide into the heme pocket of CytC is not a simple task because in CytC, as was said above (see Introduction), the heme is six-coordinate though its axial ligand, sulfur of M80, and is a weak ligand compared to distal ligand His18. Thus, there is no free space for peroxide in the heme pocket. Indeed, there is no crystal structure with peroxide or at least a water molecule in the heme pocket, but we found a crystal structure of yeast CytC with K72A replacement that contains two water molecules in the heme pocket [20] so peroxide might be, in principle, placed in the heme pocket (Figure A1 in Appendix A).

**Figure 5:**
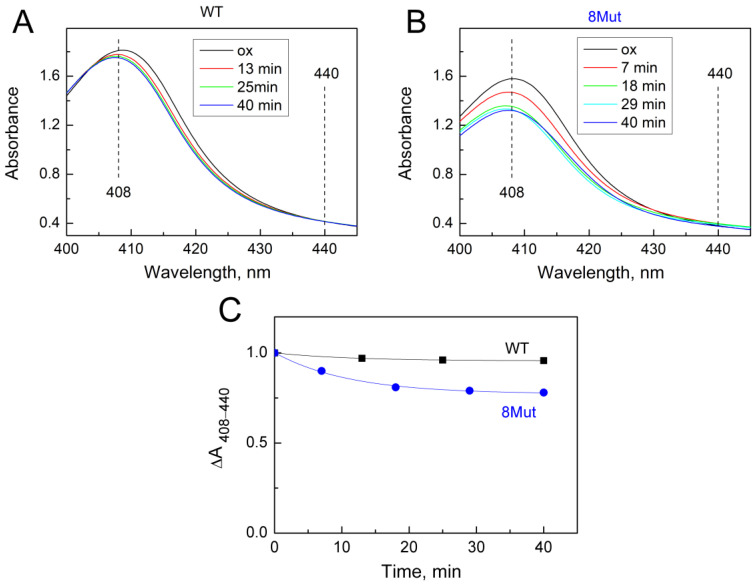
Changes in the absolute spectrum in the Soret region induced by 200 µM H_2_O_2_: (**A**)—WT CytC (17 µM), after adding H_2_O_2_ (200 µM) spectra were recorded at time intervals shown in the box: after 13 min (red), 25 min (green), and 40 min (blue); (**B**)—8Mut CytC (14 µM), after adding H_2_O_2_ (200 µM) spectra were recorded at time intervals shown in the box: after 7 min (red), 18 min (green), 29 min (light blue), and 40 min (blue). Spectra were measured in the basic buffer pH 7.6 as described in Materials and Methods. Spectrum (ox, black)—CytC (WT or 8Mut) completely oxidized by the substoichiometric amount of ferricyanide. (**C**)—The kinetics of the decrease in spectral changes in WT CytC (black curve) and 8Mut (blue curve) in the Soret region induced by 200 µM H_2_O_2_ based on the spectra presented on panels A and B. Each point corresponds to the differences ΔA_408–440_ in the amplitudes of the spectra monitored at time intervals after peroxide addition that are shown on the “X” axis. For convenience of comparison of WT with 8Mut, absorbance differences are presented as parts of the amplitude of the oxidized spectrum before addition of peroxide, which was taken equal to “1”.

**Figure 6:**
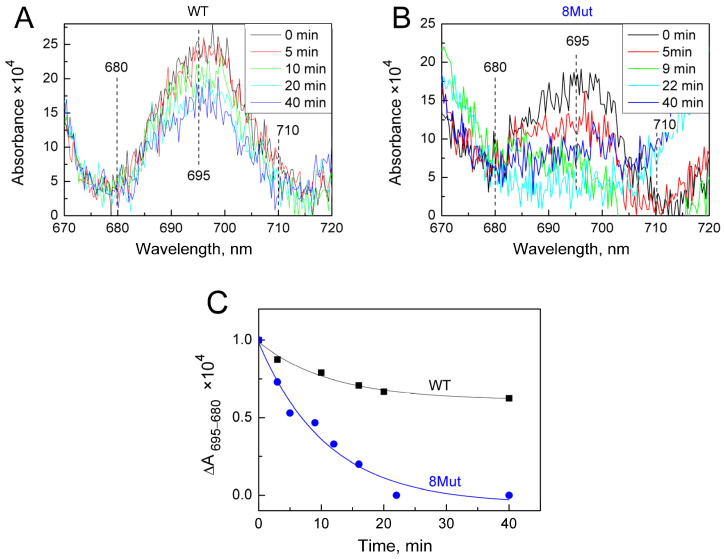
Changes in the absolute spectrum in the longwave region around 695 nm band induced by 200 µM H_2_O_2_: (**A**)—WT CytC (17 µM). Spectra were recorded at time intervals after addition of peroxide shown in the box: after 5 min (red), 10 min (green), 20 min (light blue), and 40 min (blue); (**B**)—8Mut CytC (14 µM), spectra were recorded at time intervals after addition of peroxide shown in the box: after 5 min (red), 9 min (green), 22 min (light blue), and 40 min (blue). The initial oxidized spectra (black) are marked as “0” min, other conditions as in Figure 5. (**C**)—The kinetics of the decrease in spectral changes in WT CytC (black curve) and 8Mut (blue curve) in the longwave region around 695 nm band induced by 200 µM H_2_O_2_ based on the spectra presented on panels A and B. Each point corresponds to the amplitude of the spectrum monitored at time intervals after peroxide addition that is shown on the “X” axis and calculated as absorbance difference at 695–680 nm. For convenience of comparison of WT with 8Mut absorbance differences are presented as parts of the amplitude of the oxidized spectrum before addition of peroxide, which was taken equal to “1”.

As was said above, the changes induced by the H_2_O_2_-molecule in the heme pocket and the state of the Fe…S(M80) bond can be indicated spectrally following spectral changes in the Soret region and CT band at 695 nm. Indeed, we found that peroxide itself can modulate the structure of the heme pocket by inducing the mobility of the Fe…S(M80) bond and thus making its binding with heme iron easier. Yin and coworkers [21] believe that rupture of the bond is absolutely necessary to stimulate the peroxidase activity of CytC because this frees up space in the heme pocket for binding peroxide to heme iron. The authors observed that after 20 min incubation of CytC with 0.5 mM H_2_O_2_, Soret and 695 nm bands were disrupted and mass-spectrometry revealed the modification of several amino acid residues as a result of self-oxidation. This modified CytC had enhanced peroxidase activity and is considered by the authors as an “active peroxidase” form. Taking into account these data, we also incubated CytC WT and 8Mut, which demonstrated maximal peroxidase activity (see Table 1) with 0.2 mM H_2_O_2_ at the conditions of our experimental measurements. The results are presented in Figure 5 and Figure 6. Almost no spectral changes in the Soret (Figure 5A) and 695 nm (Figure 6A) regions were observed in WT after 10 min (time range for monitoring the kinetics of peroxidase reaction—see Figure 1), and only after 40 min incubation was the amplitude of the 695 nm band decreased by ca 30% (Figure 6A,C). In contrast, incubation with peroxide causes significant changes in the spectrum of 8Mut CytC. If the amplitude of the Soret band after 40 min incubation with 200 µM H_2_O_2_ is decreased only by 35% (Figure 5B,C), the 695 nm band is decreased by one-half after 10 min and disappears after 20 min incubation with peroxide, indicating that the Fe…S bond is broken (Figure 6B,C).

## 3. Discussion

The most important additional function of CytC, which allows this protein to participate in many signaling pathways of the cell and even determine its future fate, is the proapoptotic function, which, in turn, depends on peroxidase activity, which in itself is insignificant, but is enhanced under the influence of external signals, as discussed in the Introduction.

Our previous article [17] described in sufficient detail the principles of constructing the three studied CytC mutants, which made it possible to remove CytC from participating in the ETC, but retain its ability to quench ROS, in particular superoxide. In short, key lysine residues in UBS (five in 8Mut, four in 5Mut, and one in 2Mut) were replaced by glutamate residues, and to compensate for the charge at the periphery of the protein molecule, several glutamates were replaced by lysine (constructions are presented in abbreviations), thus shifting the total dipole moment of the molecule. It was shown that [17], as a result of the transformations, 5Mut and 8Mut turned out to be completely inactive, while 2Mut retained 25% of its activity in the reaction with CcO. Despite significant differences in mutant design and activity in ETC, they all showed a number of similar changes compared to WT. First of all, peroxidase activity increased significantly in all mutants by 4–6 times. The maximum increase of 6 times was observed in 8Mut and the minimum increase of 4 times in 5Mut (Table 1). In the absence of such well-known stimulators of peroxidase activity like CL [13], we tried to find out what could cause such an acceleration of peroxidase activity. To exhibit peroxidase activity, peroxide must at least bind to heme iron, so the reason for the increase in peroxidase activity could be a change in reactivity with respect to the binding of exogenous ligands, which, according to [27,28,29], change structural stability, chemical reactivity, and physiological behavior of horse CytC. It is almost impossible to study the binding of peroxide to CytC based on the spectral changes it causes since they are small, so cyanide, the best-known exogenous ligand of heme iron, was chosen. Indeed, it turned out (Figure 2) that the formation of the cyanide complex with ferricytochrome *c* in all mutants occurs approximately twice as fast as in WT, so that in this aspect, the mutants were similar to each other and differed significantly from WT. Hence, the high peroxidase activity of the mutants is probably explained by the more rapid binding of peroxide to heme iron. The observed slight differences in the rate of formation of the cyanide complex (the fastest in 8Mut τ 3.3 min and the slowest in 5Mut τ 3.9 min (Figure 2B)) corresponded to the kinetic differences in the peroxidase activity of the mutants (Table 1).

Interesting data were also obtained when studying the effect of mutations on the degradation of the cyanide complex with the reduced form of CytC using the MCD method. As mentioned earlier, it is not easy to obtain such a complex due to the “rigidity” of the structure of reduced CytC, which does not leave space in the heme pocket for the placement of the ligand. Of note, this is why CytC, unlike CcO, cannot bind oxygen, and CytC purified from the reducing agent can remain frozen in a reduced state for months. Because of these difficulties, according to the recommendation of [26], a complex of ferrocytochrome *c* with cyanide is obtained by reducing heme iron with dithionite in the cyanide complex of oxidized CytC under alkaline conditions. But even under such conditions, the complex itself immediately begins to disintegrate, forming at the final stage the free form of ferrocytochrome *c*. The MCD method was used to study the cyanide complex for the first time, so the data are new and valuable. For the first time, it was shown (Figure 3) that mutations that “facilitated” the formation of the cyanide complex with oxidized CytC slow down the decomposition of the complex with the reduced form of cytochrome, which is evident at the spectral level (Figure 3A), in the curves of the changes in the MCD signal at 550 nm, which was reconstructed manually on the basis of spectral data (Figure 3B); the curves were recorded in kinetic mode, tracking the decrease in the MCD signal at 555 nm for 8Mut and WT (Figure 3C) and on the content of the CytC^2+^-CN complex in samples at each moment of the recorded MCD spectra, which was calculated using the Graph Work program (Table 2). It can be assumed that mutant cytochromes have a more “free” conformation, which facilitated the reaction of oxidized CytC with cyanide, and are temporarily stabilized and provide the ligand with greater freedom of placement in the heme pocket.

It was noted in the early studies [30] that a key role in increasing the “mobility” of the CytC structure is played by Met80, or more precisely the distance of the coordination bond of heme Fe…S(Met80). It is interesting, for instance, that acetylation of Met80 (attachment of a CH_2_-COO- group) leads to the fact that CytC begins to react with oxygen [31]. To clarify the question of how mutations affect the mobility of atoms in the CytC structure and the coordination bond Fe…S(Met80), molecular dynamics simulations of CytC WT and mutants were carried out. The standard deviation of fluctuations of individual residues (Figure 4A) and differences in isoelectric surfaces induced by mutations (Figure 4B) were analyzed. Figure 4A shows that individual residues in the cytochrome molecule of WT and mutants fluctuate significantly, deviating from the initial position. A significant increase in the mobility of residues in 8Mut should be noted. It is especially important that residues 79K and 80M in 8Mut deviate more than three times higher from the initial position compared to WT. Significant mobility of residues 84–86 is also observed in 2Mut while the flexibility of the residues in 5Mut in this region is not that great and does not deviate from WT. Residues 70–86 form the so-called Ω-loop, one of the most flexible parts in the CytC structure, and residues 79K and 80M (axial ligand of heme iron) especially play an important role in CytC interaction with exogenous ligands, including peroxide. It is worth noting that the increased mobility of the residues in the Ω-loop correlates directly with the increase in peroxidase activity of CytC, which can be ranked as 8Mut > 2Mut > 5Mut > WT. The increased flexibility of the Ω-loop together with the diverse isoelectric surface of the protein globule revealed in all mutants must obviously facilitate the interaction of heme with peroxide and other external ligands and increase peroxidase activity.

Also noteworthy is the general increase in atomic mobility of 8Mut and 5Mut in the region of residues 42–56, with the maximum fluctuations observed at residues 45, 52, and 56 that three–four times exceed the deviations in WT. In this regard, it is worth recalling (see Introduction) that residues Asn52 and Pro44, the residues with increased mobility in 8Mut, belong to the so-called site C, which according to one of the generally accepted hypotheses binds to cardiolipin and triggers proapoptotic peroxidase activity [13]. The preservation of the Fe…S(M80) bond indicated by the presence of the CT 695nm band in the absolute spectra of WT and the mutants observed earlier [17] is also questionable upon monitoring the peroxidase activity. As shown in Figure 5 and Figure 6, incubation with peroxide induces significant changes in the structure of the heme pocket and results in the complete disappearance of the 695 nm band in 8Mut within 20 min.

Based on the results of both this work and the previous one, attention is drawn to the fact that 5Mut deviates to the greatest extent in its physico-chemical properties from both WT and the other two mutants. Deviations are expressed both in an increased tendency to aggregation of molecules, changes in the CD spectrum and molar extinction at 550 nm (according to [17]) and changes in the isoelectric surface, revealed by molecular dynamics modeling, which are more dramatic as in other mutants and WT. We attribute such deviations to the general charge imbalance in this mutant, where four K/E substitutions are compensated by a single E/K substitution, and a change in the dipole moment of the molecule. The resulting drastic changes in the physico-chemical properties of CytC reflect the important role played by electrostatic interactions in the overall stabilization of the CytC structure.

Furthermore, analyzing the data obtained in the article, what is really surprising is not the differences between individual mutants, but a certain “coherence” in comparison with WT, manifested in approximately the same acceleration of the peroxidase reaction and the formation of the cyanide complex with oxidized CytC, and approximately the same slowdown in the decomposition of the cyanide complex with a reduced form of CytC, characteristic for all mutants regardless of their structure. It seems that the mentioned changes in activity are provided by only one K72E substitution, which is present in all three mutant forms, and the remaining mutations provide only small fluctuations. However, it is worth noting that this is the only lysine substitution in the so-called red Ω-loop (70–85), which plays a very important role in the CytC structure and functions [32]. Structural rearrangements of globular proteins, which include CytC, occur due to weakly structured and conformationally flexible loops. The heme crevice loop (red Ω-loop) formed by residues 70–85, including heme ligand Met80, is the most conserved and the most flexible segment of CytC primary structure. The peroxidase activity of CytC remains weak as long as the Met80 residue remains coordinated with heme iron. As has already been discussed in detail above, when the Fe…S(Met80) bond is broken, a free space is formed for the binding of hydrogen peroxide and other ligands, as a result of which the peroxidase reaction is activated. The significance of K72 replacement for the structure of heme pocket was revealed by the features elucidated in the 3D structure of mutant CytC K72A from yeast obtained by McClelland et al. with a resolution of 1.45 Å [20]. Figure A1 in Appendix A represents crystal structures of six-coordinated CytC WT from horse heart (PDB ID: 1HRC, green) and yeast CytC with a K72A mutation (PDB ID: 4MU8, gray), which are aligned along α- helices and superimposed, as seen in the heme iron of the K72A mutant, which is five-coordinated with its 6-th axial ligand sulfur (M80) turned aside. In the other version presented in [20], sulfur is replaced by water as is observed in other classic high-spin hemoproteins like cytochrome oxidase. The authors reported the presence of an internal water channel. It is likely to suppose that the mutation of Lys72 residue to Glu in CytC from horse heart analogous to K72A in yeast contributed to an increase in the mobility of Met80, an increase in the distance between Fe and S(Met80) [20], and the opening of a water channel through which hydrogen peroxide enters the heme cavity and activates the peroxidase reaction. However, the authors of [20] also emphasize that in the process of evolution, animals developed steric hindrances in opening the water channel in order to prevent apoptosis.

The last but not least thing we would like to discuss here is the proposed new method for recording the peroxidase activity of CytC. Several methods for measuring peroxidase activity are described in the literature, the most sensitive of which use low concentrations of peroxide (25 µM) and are based on chemiluminescence induced by luminol peroxidation [3,22]. The method involves comparative assessments, but does not allow the speed to be determined quantitatively. A number of other methods use typical substrates of peroxidases like ABTS and guaiacole with known molar extinction coefficients, which make it possible to quantify the rate of substrate conversion by monitoring their peroxidation spectrophotometrically [18,19,20,21]. However, due to the difficulties of the interaction of high-potential substrates with CytC, the authors had to use very high (50–100 mM) peroxide concentrations and a rapid mixing technique, so the kinetics are difficult to compare with steady-state ones.

What is a physiologically possible peroxide concentration inside the cell is not so simple a question. A new fluorescent indicator HyPer was presented by [33] to detect H_2_O_2_ inside living cells. Later, using the same indicator, HyPer 2.2 ± 0.4 nM was reported as a close-to-normal concentration of H_2_O_2_ in the cytosol of undisturbed cells [34]. In other studies, incubation of HeLa cells with 125 µM peroxide [35] induced apoptosis in HeLa culture, but the effect was not too hard, so that 11.05% apoptosis was observed after 1 h incubation, and 28.96% apoptosis after 3 h. Later incubation of fibroblasts with 200 µM peroxide for half an hour induced apoptosis, which could be reversed by antioxidant SkQ [36]. To say, briefly, the 200 µM peroxide that we used to measure the peroxidase activity of CytC cannot be considered very toxic, but it represents the upper limit of concentrations physiologically acceptable for cells.

At the same time, according to our observations, 10 min incubation (time range of monitoring the peroxidase activity) of 8Mut CytC with 0.2 mM peroxide was enough to trigger a decrease in the Soret (which actually did not exceed 5%, Figure 5B,C) and a two times decrease in the 695 nm band (Figure 6B,C). The decrease in the 695 nm band is important because reflects significant changes in the Fe…S(M80) bond induced by peroxide in 8Mut CytC. At the same time, even 40 min incubation of WT CytC with 0.2 mM H_2_O_2_ did not cause much disturbance and the decrease in the amplitude of the 695 nm band does not exceed 35% (Figure 5A,C). The destabilizing effect of peroxide in relation to CytC is facilitated by the surface location of heme iron, while other hemoproteins, for example, CcO, are much more resistant to it. The effect of peroxide should undoubtedly be taken into account when choosing a method for measuring peroxidase activity; therefore, even under conditions of rapid mixing, 50–100 mM of peroxide added to CytC in [19] can lead to uncontrolled disturbances in the protein structure. The reported rates of guaiacol peroxidation by CytC from horse heart (0.13 s^−1^) are approximately an order of magnitude lower than the rate of ABTS oxidation (1.77 s^−1^), which the authors do not comment on [19]. But even in those experiments where moderate concentrations of peroxide were used (0.1–0.5 mM), problems arose, since the oxidation of ABTS leads to the formation of a stable radical cation ABTS+, the concentration of which is actually measured. If the ABTS+ concentration is artificially reduced due to the radical quenching properties of CytC, the use of such a substrate may cause errors in reaction rate measurements. The other study by Yin et al. [21] considers self-oxidation of CytC induced by H_2_O_2_ as a necessary condition for the transformation of CytC to “active peroxidase”. By the mass-spectrometry method, the authors identified Y67 and K72/73 residues as possibly oxidized/carbonylated. At the same time, it should be noted that peroxidase activity of modified CytC and native protein is not the same thing. All kinetic traces presented in this study have a pronounced lag phase, and steady-state phase, and end with the deceleration of the reaction rate indicating deactivation of the protein. As seen in Figure 1, the kinetic traces in the current study show a linear dependence on time without any lag-phase or deactivation. This suggests that the true peroxidase activity of native CytC of WT and mutant forms is being measured. A small lag phase is observed only in Mut8. We have associated it for some time as being necessary for the binding of peroxide to heme iron, but we also cannot exclude some covalent modifications induced by H_2_O_2_ and mentioned in [21], especially taking into account that the peroxidase activity of 8Mut is higher compared to other mutants.

Thus, we suggest a new method for recording the peroxidase activity of CytC under gentle native conditions practically excluding protein modification caused by peroxide using a ferro-ferricyanide buffer as a reaction substrate, which allows flexible adjustment of the medium’s Em value to specific conditions. In this case, the negative charge of ferro/ferricyanide ensures optimal interaction with CytC, which has been well studied [37]. Previously, we successfully used this method to measure the peroxidase activity of CcO under aerobic conditions at a ferro/ferri ratio of 1:1 (Em +420 mV), which allows peroxide to win the competition for the electron over oxygen [7,38]. Considering the lower value of Em +260 mV CytC from the horse heart [39], the optimal ferro/ferri ratio was 6:1 (Em +370 mV). Under these conditions, the peroxidation of ferrocyanide catalyzed by CytC WT, and especially by the mutant forms, significantly exceeds the rate of its oxidation by the peroxide itself (Figure 1), which should be taken into account when calculating the rate. The reaction is easily monitored spectrophotometrically, and an increase in time compensates for the small value of the molar extinction of the oxidation product—ferricyanide (1040 M^−1^ cm^−1^). It is also important that not very high concentrations of peroxide (0.2 mM) can be used.

The closest method for recording the peroxidase activity of CytC, using relatively low concentrations of H_2_O_2_ (0.1–0.5 mM), is described in [18]. The data from this work allowed us to select parameters for optimal recording of CytC peroxidase activity (pH 7.1, CytC concentration—3 µM—within these limits, the rate linearly depends on the CytC concentration), without engaging in a more detailed study of the reaction. Kim and co-workers used ABTS as a substrate and monitored its oxidation product, the stable radical cation ABTS+. As the authors themselves showed [18], the reaction is inhibited by typical radical quenchers: formate, azide, and ethanol. In the same work [18], the authors observed the almost complete (96–97%) inhibition of ABTS peroxidation with free iron chelators 5 mM EDTA and 0.1 mM deferoxamine (DFX). When recording the peroxidase reaction, we specially added 0.2 mM EDTA to the buffer to exclude the influence of impurity iron, which was not undertaken by other authors. Moreover, it was specifically verified that 5 mM EDTA and 0.1 mM DFX, as well as formate and ethanol, do not affect ferrocyanide peroxidation.

## 4. Materials and Methods

Components for the culture media and buffer solutions for chromatography and electrophoresis (AppliChem, Darmstadt, Germany), ampicillin, CytC from horse heart (Sigma, Schnelldorf, Germany), *Xho* I restriction endonuclease (Promega, Madison, WI, USA), *Bam*H I restriction endonuclease (New England Biolabs Inc., Ipswich, MA, USA), *Pfu*-DNA polymerase, and *T4*-DNA ligase (Fermentas, Pabrade, Lithuania) were used in this study. Distilled water was additionally purified on a Milli-Q system (Millipore, Burlington, MA, USA). Potassium ferro- and ferricyanide, sodium dithionite, 2,2′-Azino-bis(3-ethylbenzthiazoline-6-sulfonic acid (ABTS), guaiacole, and hydrogen peroxide were from Sigma-Aldrich (USA). Hydrogen peroxide solution (about 30%) was kept at 4 °C, before the experiments the concentration was checked spectrophotometrically using molar extinction coefficient ε_240_ = 40 M^−1^ cm^−1^ [40]. The concentration of CytC was determined from the difference absorption spectra (dithionite reduced vs ferricyanide oxidized) using molar extinction coefficient ∆ε_0550–540_nm = 18.7 mM^−1^ cm^−1^ [41]. pH-Buffers and EDTA (ethylenediaminetetraacetic acid) were from Amresco (Solon, OH, USA).

### 4.1. Mutant Genes Construction

Mutation of horse heart CytC gene within expression plasmid vector pBP(CYC1/CYC3) was performed by site-directed mutagenesis as recommended using the QuikChange Mutagenesis Kit (Stratagene, La Jolla, CA, USA) [42]; the procedure is described in more detail in [17]. The nucleotide sequences of the mutated CytC genes were determined using an ABI Prism 3100-Avant Genetic Analyzer automated sequencer (Applied Biosystems, Beverly, MA, USA). Selected mutant genes were cloned into expression vector pBP(CYC1/CYC3), modified for the expression of horse heart CytC.

### 4.2. Expression of the Mutant Genes of CytC, Protein Isolation, and Purification

Mutated genes were expressed in *E. coli* strain JM-109 grown at 37 °C for 22–24 h in vigorously stirred liquid medium SB containing ampicillin (0.2 mg/mL) [16,42]. The cells were harvested by centrifugation at 4000× *g* (4 °C) for 20 min. The cell pellet was resuspended in buffer (25 mM Na-Pi, pH 6.0) containing 1 mM NaN_3_ and frozen at −20 °C for 20–30 min, after which it was subjected to high-pressure disintegration in a French Press. The resulting homogenizate was centrifuged at 90,000 G for 20 min. The protein was purified using an “AKTA FPLC” liquid chromatographic system (GE Healthcare, Chicago, IL, USA) as described previously [16,42]. The cell extracts prepared as described above were applied on an MP HS 10/10 cation-exchange column (BioRad, Hercules, CA, USA) equilibrated with the same buffer (25 mM Na-Pi, pH 6.0, 1 mM NaN_3_). The extracts containing mutant proteins with four or more substitutions were dialyzed against the same buffer before chromatography. CytC was eluted by the same buffer with linear gradient of 1 M NaCl at the rate of 3 mL/min. The fractions eluted from MP HS were analyzed spectrophotometrically and by SDS-electrophoresis in 12% Tris-Tricine PAAG. The fractions containing cytochromes *c* were dialyzed against the buffer for absorption chromatography (10 mM Na-Pi, pH 7.0, 1 mM NaN_3_) and applied on a column with hydroxyapatite CHT-I (BioRad, Hercules, CA, USA). CytC was eluted in a 0.5 M Na-Pi (pH 7.0) gradient at the rate of 2 mL/min. The mutant CytC 8Mut was further purified by gel filtration on Superdex-200 10/300 column (GE Healthcare, Chicago, IL, USA) equilibrated with 50 mM Na-Pi (pH 7.2), 150 mM NaCl at the rate of 0.5 mL/min. The purification degree of CytC was estimated spectrophotometrically and by SDS-electrophoresis. The fractions containing 95% pure proteins were combined and the samples were dialyzed twice against 10 mM ammonium carbonate, pH 7.9. The proteins were lyophilized on ALPHA I-5 and stored at −20 °C.

### 4.3. Spectrophotometric Assays

Spectrophotometric assays were carried out in a standard semi-micro cuvette (Hellma, Müllheim, Germany) with blackened side walls, 10 mm light pathway, and the slit width used was 1.5 nm. Absolute spectra of CytC^3+^-CN complex were recorded in 50 mM Hepes/Tris buffer, pH 7.6, 0.1 mM EDTA, and 50 mM KCl at speed of 2 nm/s, in a double-beam spectrophotometer Cary 300 Bio (Varian, Palo Alto, CA, USA). Since preparations of CytC were at different states of oxidation, to standardize the conditions, substoichiometric amounts of ferricyanide were added to the samples and the completeness of oxidation was controlled spectrally.

Kinetic measurements of CytC peroxidase activity were made on the spectrophotometer SLM Aminco DW-2000 (SLM Instruments, Wixom, MI, USA) in a dual-wavelength mode in 20 mM MOPS pH 7.1, 50 mM KCl, 0.2 mM EDTA buffer, following peroxidation of ferrocyanide by absorption difference at 420 nm vs. 500 nm reference. The rate values in Table 1 were averaged from 3–4 recordings with indication of the deviations.

### 4.4. MCD Spectroscopy

MCD spectra were recorded in a 50 mM K-Pi/tris buffer (pH 8.7), with equipment assembled in the laboratory of Hemoproteins of A.N. Belozersky Institute of Physico-Chemical Biology, Lomonosov Moscow State University on the base of a Jobin Yvon Mark V auto-dichrograph (France, Paris) supplied with a permanent magnet (effective magnetic field ~0.7 T for a 1 cm cell). A computer program Graph Work was also developed in the laboratory to provide the control of the Instrument, data collection, and results processing.

MCD spectra were recorded in a wavelength range of 530–565 nm at 25 °C in a quartz cuvette with optical pass 10 mm and total volume ca 400 μL.

Concentration of CytC in the sample was around 25 µM. KCN stock solution 10 M was added to CytC to final concentration 100 mM and after 5 min incubation dithionite powder was added and registration began with time control. All spectra were normalized to protein concentration measured after experiment.

### 4.5. Molecular Dynamics

Molecular dynamics was applied to CytC WT and mutant forms based on CytC WT structure PDB ID 1HRC. Mutations were incorporated into the structure using PyMol software (PyMol version 2.5.0 was used license information build on open source) similar to [43]. MD calculations were carried out in the Gromacs-2023 software (Gromacs 2023.5, open source software) package using Charmm27 force field. A protein molecule with heme was placed in a 5 nm × 5 nm× 5 nm cell and optimized and immersed in physiological conditions: an aqueous solution with 50 mM NaCl pH 7.0 (which corresponds to 3500 water molecules and 15 Na^+^, Cl^−^ ions). At such conditions, aspartate and glutamate residues are deprotonated, and lysine and arginine are protonated. All calculations of molecular dynamics were carried out at an operating temperature 300 K. Preliminary molecular dynamic calculations of 100–200 ns duration with a temperature control according to Berendsen with a step of 1 fs, a recording period of 1 ps were used to equilibrate the system then followed by 350 ns stochastic dynamics calculations consistent with the canonical ensemble and correctly reflecting the properties of the system [44]

### 4.6. Isoelectric Surfaces

Isoelectric surfaces were built from electrostatic potentials calculated similarly as described in [17] using the Poisson–Boltzmann equation in the ProKSim program [45] developed at the Department of Biophysics of Moscow State University.

## 5. Conclusions

The introduction of site-directed mutations in UBS of CytC and a shift in the dipole moment of the molecule led to significant functional changes in the protein, as well as some changes in physico-chemical properties, while generally maintaining the secondary structure of CytC, which was discussed in detail in our previous article [17]. The current article shows that these mutations increase the flexibility of the residues in the Ω-loop (70–85), which in turn leads to a weakening of the coordination bond between the Fe atom of hemoporphyrin and sulfur of Met80 Fe…S(Met80) and, as a consequence, contributes to an increased tendency to bind external ligands and accelerate the peroxidase-like activity of CytC by 5–6 times. It is noteworthy that changes in activity are provided by just one substitution K72E, which is present in all three mutant forms. K72, the only substituted lysine, is part of the so-called Ω-loop (70–85), which indicates the important functional role of K72 and the Ω-loop in activating the peroxidase activity of CytC. A new simple method for recording the peroxidase activity of CytC has been proposed, using a ferro/ferricyanide buffer in a ratio of 6:1 as a substrate.

## Figures and Tables

**Table 1:** Peroxidase activity of CytC WT and its mutant variants (standard deviations are shown). The CytC concentration in the samples was 3 µM. Other conditions as in Figure 1 are given in the caption to Figure 1.

Type of CytC	WT	2Mut	5Mut	8Mut	Control without CytC
µM ferri/min	1.7 ± 0.1	5.6 ± 0.03	5.0 ± 0.5	6.6 ± 0.2	0.77 ± 0.11
(TNs-control)/[c]	0.31 min^−1^	1.61	1.41	1.95	

**Table 2:** The percentage content of the cyanide complex at each time point after the reduction of cytochrome *c* with dithionite.

Time, min	0.3	1.2	2.1	2.9	3.7	4.5	5.3	6.1	6.9
Mut8-CN	97	86.5	65.1	52	44	32	28.7	23	19
WT-CN	95	75	59	48.5	38	30.6	26	20	16
Time, min	7.7	8.5	9.3	10.1	10.9	11.7	12.5	13.3	14.1
Mut8-CN	18	11.9	10.7	9.4	9.4	6.8	5	1.5	0.5
WT-CN	13	10	9.6	7.0	3.9	3.9	3	0.5	0.04

## Data Availability

All raw data are available from corresponding authors under reasonable request.

## Abbreviations

ABTS—2,2′-Azino-bis(3-ethylbenzthiazoline-6-sulfonic acid; CytC—cytochrome *c*; CytC 2Mut, 5Mut and 8Mut—cytochrome *c* with substitutions E69K/K72E, K8E/E69K/K72E/K86E/K87E and K8E/K27E/E62K/E69K/K72E/K86E/K87E/E90K, respectively; complex *bc1*—ubiquinol:cytochrome *c* oxidoreductase; CcO—cytochrome *c*-oxidase, ferrocytochrome *c*:oxygen oxidoreductase; ETC—electron transport chain; IMS—intermembrane space of mitochondria; MCD—magnetic circular dichroism; ROS—reactive oxygen species; UBS—universal binding site; WT—wild type, RMSF—root mean square fluctuations, CT—charge transfer band.
